# Evaluating Dog Bite‐Induced Facial Trauma: A Study From a Single Tertiary Care Center

**DOI:** 10.1002/oto2.70233

**Published:** 2026-04-20

**Authors:** Andrew J. Rothka, Marly Aziz, Katelyn P. K. Nguyen, F. Jeffrey Lorenz, Heather K. Schopper, Jessyka G. Lighthall

**Affiliations:** ^1^ Department of Surgery University of North Carolina Chapel Hill North Carolina USA; ^2^ College of Medicine The Pennsylvania State University Hershey Pennsylvania USA; ^3^ Department of Otolaryngology–Head and Neck Surgery Penn State College of Medicine Hershey Pennsylvania USA

**Keywords:** dog bite, dog bite injuries, facial trauma

## Abstract

**Objective:**

Analyze demographic and clinical factors for patients presenting with dog bites to the face, head, or neck.

**Study Design:**

Retrospective cohort.

**Setting:**

Single tertiary care, level 1 trauma center.

**Methods:**

A retrospective chart review identified patients presenting for facial dog bite injuries from 2012 to 2023.

**Results:**

There were 906 patients with facial dog bites and 2061 injuries. Patients ranged from 0 to 91 years old with a median of 7 years, and 50.6% were female. The 2 most common causes for injury were playing with the dog (32.0%) or an unprovoked attack (17.3%). Family dogs were responsible for 61.3% of injuries. Pitbulls (22.4%), Labradors (10.5%), and German Shepherds (7.0%) were the most identified breeds. The cheeks (25.9%), lips (20.8%), and nose (9.0%) were most injured. Hospital admission was required in 17.0% of patients. Regarding management, 16.9% required operative repair, 65.9% were repaired at bedside, and 17.9% were managed conservatively. There were 878 patients prescribed intravenous or oral antibiotics (96.9%) with amoxicillin‐clavulanate most frequently prescribed (62.7%). Of the 138 patients bit by dogs with outdated or unknown rabies vaccination status, 35.5% (n = 49) received a rabies vaccination, and 34.8% (n = 48) received rabies immunoglobulins. Of the 254 patients not current on tetanus vaccination, 78.7% (n = 200) received a booster.

**Conclusion:**

Dog bites to the head and neck are common injuries, with many patients requiring hospital admission and/or repair. Understanding the demographics of these injuries is important to identify physical and socioeconomic burdens of disease, gaps in adherence to protocols/guidelines, and areas of targeted education/preventative interventions.

According to the American Veterinary Medical Association (AVMA), dog ownership in the United States increased over 40% since 1996, with roughly 89.7 million dogs belonging to 59.8 million households as of 2024.^1^ Though having a dog at home provides physical and psychological benefits, 1 study found 80.2% of dog bites occurred at home.^2^, ^3^ Approximately 4.7 million people are bitten by dogs annually in the United States.^4^ Injuries from dog bites are a public health concern since they remain one of the most common nonfatal injuries over the past few decades.^5^, ^6^

Previous studies demonstrate that dog bites, especially those to the head, neck, or face, occur more predominantly in pediatric populations.^7^, ^8^, ^9^, ^10^, ^11^, ^12^ A narrative review from 2022 reports a significant disproportion between the number of children versus adults with facial dog bite injuries.^13^ This same study found the 3 most common sites of injury are the lips, cheeks, and nose.^13^ The purpose of the current study is to evaluate patterns, demographics, and clinical risk factors for facial trauma from dog bites in patients of all ages seen at a single level 1 trauma center in Central Pennsylvania.

## Methods

A retrospective chart review identified patients presenting to the Penn State Milton S. Hershey Medical Center for facial dog bite injuries between January 1, 2012, and December 31, 2023. The following data was collected, if available: age at visit, date of incident, sex, primary location of injury, secondary location of injury, tertiary location of injury, number of injuries, hospital admission (yes/no), type of repair (bedside/operative/none), tetanus vaccination administration, rabies vaccination administration, rabies immune globulin administration, antibiotics given, dog breed, relation to dog (family/other), what patient was doing at time of injury, dog vaccination status, and behavioral comorbidities of patient. This study was reviewed and approved by the Pennsylvania State College of Medicine Institutional Review Board (IRB) (Study ID# 24260).

### Statistical Analysis

Microsoft Excel was utilized for data collection and analysis. Descriptive statistics including mean, median, and percent change were calculated.

## Results

A total of 906 patients with facial trauma secondary to dog bites were identified. Over the study period, a total of 2061 injuries were recorded. Incidence of injuries and patients were listed in Table 1 along with percent change in each from the year prior. 2019 was the calendar year with the largest number of injuries (n = 268/2061, 13.0%) and patients (n = 114/906, 12.6%). The greatest change in number of injuries and patients occurred when comparing 2020 with 2019. There was a 22.4% decrease in the number of injuries and a 25.4% decrease in the number of patients from 2019 to 2020.

**Table 1:** Incidence of Dog Bite Facial Trauma by Year

Year	Number of injuries recorded (% change)	Number of patients (% change)
2012	69	31
2013	122 (+76.8%)	59 (+90.3%)
2014	136 (+11.5%)	51 (−13.6%)
2015	173 (+27.2%)	48 (−5.9%)
2016	137 (−20.8%)	54 (+12.5%)
2017	120 (+12.4%)	54 (0.0%)
2018	188 (+56.7%)	84 (+55.6%)
2019	268 (+42.6%)	114 (+35.7%)
2020	208 (−22.4%)	85 (−25.4%)
2021	178 (−14.4%)	95 (+11.8%)
2022	227 (+27.5%)	120 (+26.3%)
2023	235 (+3.5%)	111 (−7.5%)

To analyze the impact of time of year, each year was divided into quarters (January‐March, April‐June, July‐September, and October‐December). January to March had 178/906 patients (19.7%), April to June had 246/906 patients (27.2%), July to September had 244/906 patients (27.0%), and October to December had 238/906 patients (26.3%).

Patients ranged from 0 to 91 years old with a median age of 7 years. The most common age ranges were 0 to 4 years (n = 309/906, 34.1%) and 5 to 9 years (n = 219/906, 23.9%). The distribution of age ranges by year was listed in Table 2. In our cohort, there was a slight female predominance (n = 458/906, 50.6%).

**Table 2:** Distribution of Patient Age by Year

Age range	Total	2012	2013	2014	2015	2016	2017	2018	2019	2020	2021	2022	2023
0‐4	309	8	22	24	21	24	22	26	40	31	22	39	30
5‐9	219	7	8	10	15	11	14	24	31	18	21	33	27
10‐14	112	4	7	3	5	7	7	12	14	11	15	10	16
15‐19	44	1	8	0	1	1	2	3	6	4	7	5	6
20‐24	30	2	3	2	1	1	1	4	2	5	4	2	3
25‐29	39	2	3	2	3	4	3	2	2	1	3	8	5
30‐24	16	1	0	2	0	1	2	0	1	1	2	3	3
35‐39	25	1	1	2	0	1	2	5	4	1	5	1	2
40‐44	19	1	1	2	1	0	1	1	4	2	2	2	2
45‐49	18	2	1	0	0	1	0	1	1	1	4	4	3
50‐54	21	1	1	1	0	1	0	3	2	2	2	4	4
55‐59	21	1	1	2	0	0	0	1	3	3	2	4	4
60+	33	0	2	1	0	2	0	2	4	2	6	5	6

**Figure 1:**
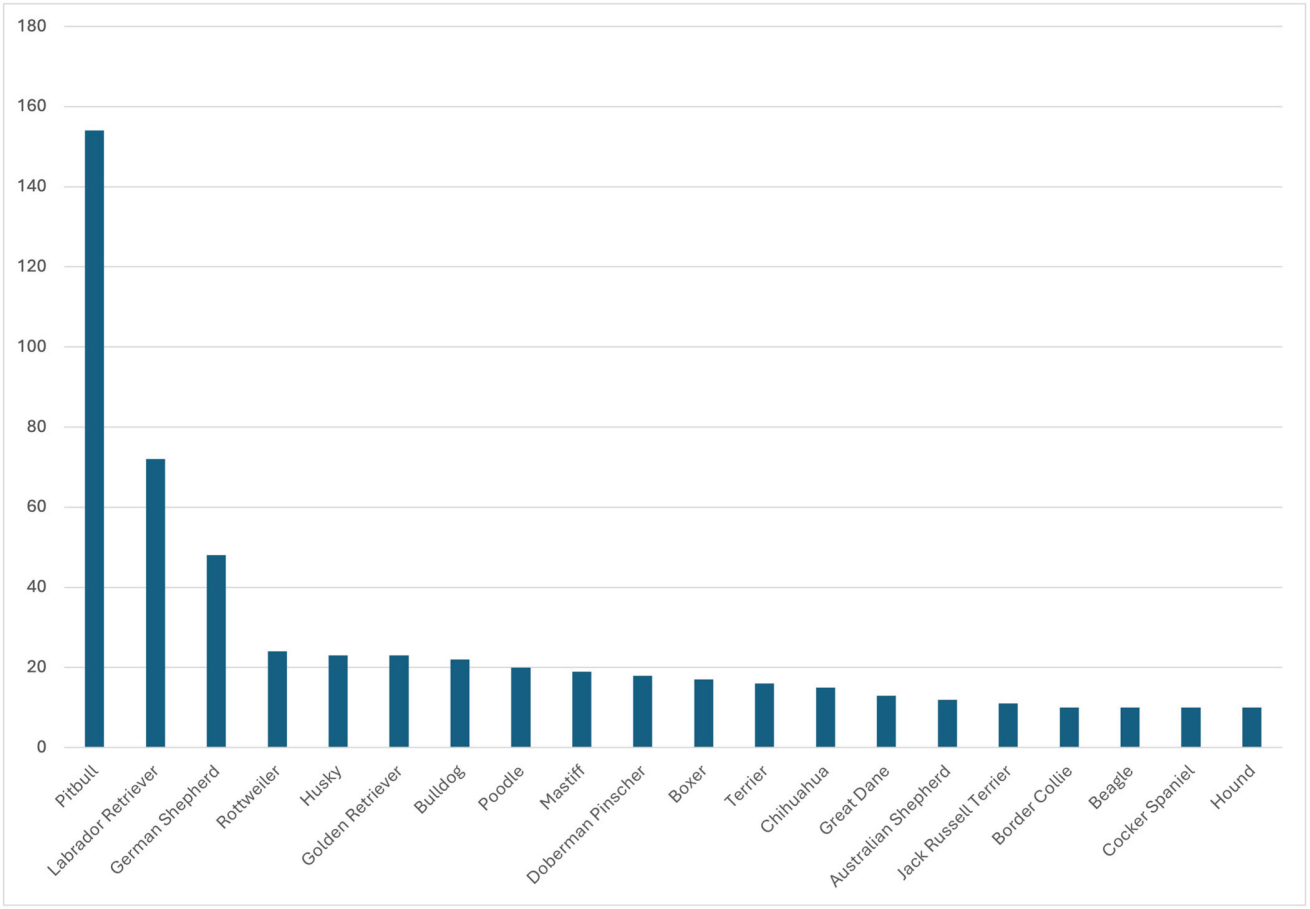
Distribution of 20 most commonly known dog breeds involved in facial trauma.

A total of 998 dog breeds (68 unique breeds) contributing to 912 dogs were documented as being involved in facial trauma. However, 31.3% of breeds were either unknown or not mentioned (n = 312/998). When breed was reported (n = 686), the most involved were Pitbulls (n = 154/686, 22.4%), Labrador Retrievers (n = 72/686, 10.5%), and German Shepherds (n = 48/686, 7.0%). Mixed breeds (eg, Goldendoodles) were separated into their respective breeds (eg, Golden Retriever and Poodle) for ease of analysis. The number of dogs of known/reported breeds were listed in Table 3. The distribution of the twenty most identified dog breeds was illustrated in Figure 1.

**Table 3:** Reported Dog Breeds Involved in Facial Trauma

**Dog breed**	Total (n) (%)
Pitbull	154 (22.4%)
Labrador Retriever	72 (10.5%)
German Shepherd	48 (7.0%)
Rottweiler	24 (3.5%)
Husky	23 (3.4%)
Golden Retriever	23 (3.4%)
Bulldog	22 (3.2%)
Poodle	20 (2.9%)
Mastiff	19 (2.8%)
Doberman Pinscher	18 (2.6%)
Boxer	17 (2.5%)
Terrier	16 (2.3%)
Chihuahua	15 (2.2%)
Great Dane	13 (1.9%)
Australian Shepherd	12 (1.7%)
Jack Russell Terrier	11 (1.6%)
Border Collie	10 (1.5%)
Beagle	10 (1.5%)
Cocker Spaniel	10 (1.5%)
Hound	10 (1.5%)
Akita	9 (1.3%)
Shih Tzu	8 (1.2%)
Boston Terrier	7 (1.0%)
St. Bernard	7 (1.0%)
Dachshund	7 (1.0%)
Alaskan Malamute	6 (0.9%)
Catahoula Leopard	6 (0.9%)
English Springer Spaniel	5 (0.7%)
German Shorthaired Pointer	5 (0.7%)
Bassett Hound	4 (0.6%)
Belgian Malinois	4 (0.6%)
Pomeranian	4 (0.6%)
Coonhound	4 (0.6%)
American Staffordshire Terrier	3 (0.4%)
Cattle Dog	3 (0.4%)
Blue Heeler	3 (0.4%)
Bloodhound	3 (0.4%)
Chow Chow	3 (0.4%)
Rat Terrier	3 (0.4%)
Collie	3 (0.4%)
Irish Setter	3 (0.4%)
Shiba Inu	3 (0.4%)
Whippet	2 (0.3%)
Pug	2 (0.3%)
Norwegian Elkhound	2 (0.3%)
Greyhound	2 (0.3%)
Chesapeake Bay Retriever	2 (0.3%)
Foxhound	2 (0.3%)
Maltese	2 (0.3%)
Yorkshire Terrier	2 (0.3%)
Weimaraner	2 (0.3%)
Corgi	2 (0.3%)
American Eskimo	1 (0.1%)
Bernese Mountain Dog	1 (0.1%)
Bichon Frise	1 (0.1%)
Newfoundland	1 (0.1%)
Eskimo Spitz	1 (0.1%)
Wolf	1 (0.1%)
Dalmatian	1 (0.1%)
English Transitive	1 (0.1%)
Rhodesian Ridgeback	1 (0.1%)
Dutch Shepherd	1 (0.1%)
Swiss Mountain Dog	1 (0.1%)
Gordon Setter	1 (0.1%)
Great Pyrenees	1 (0.1%)
Cane Corso	1 (0.1%)
Shetland Sheepdog	1 (0.1%)
Italian Water Dog	1 (0.1%)

**Figure 2:**
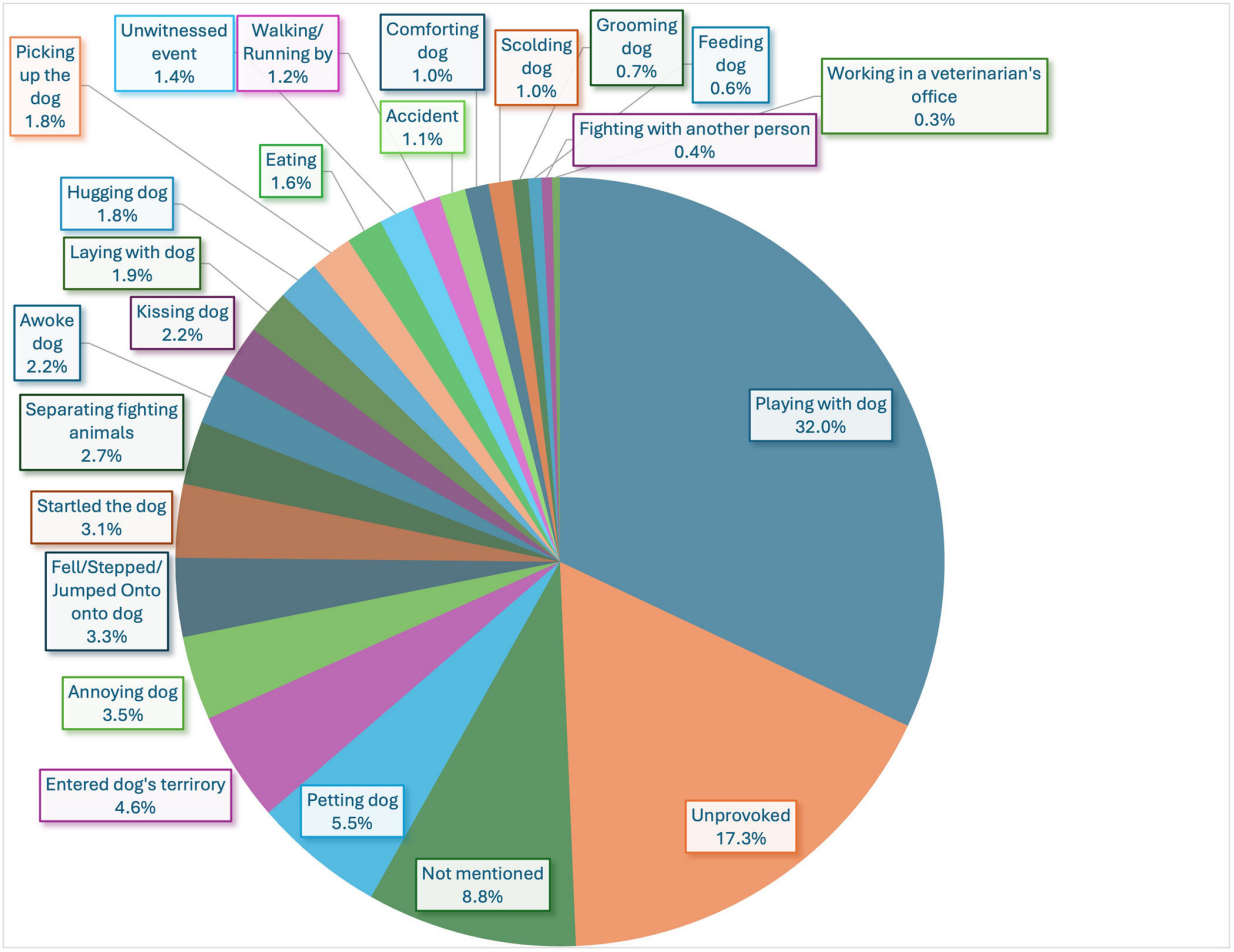
Provocation of dog bites.

The 2 most common causes for injury were playing with the dog (n = 290/906, 32.0%) or unprovoked attacks (n = 157/906, 17.3%). The full distribution of actions performed by patients thought to provoke the dog bite was illustrated in Figure 2.

**Figure 3:**
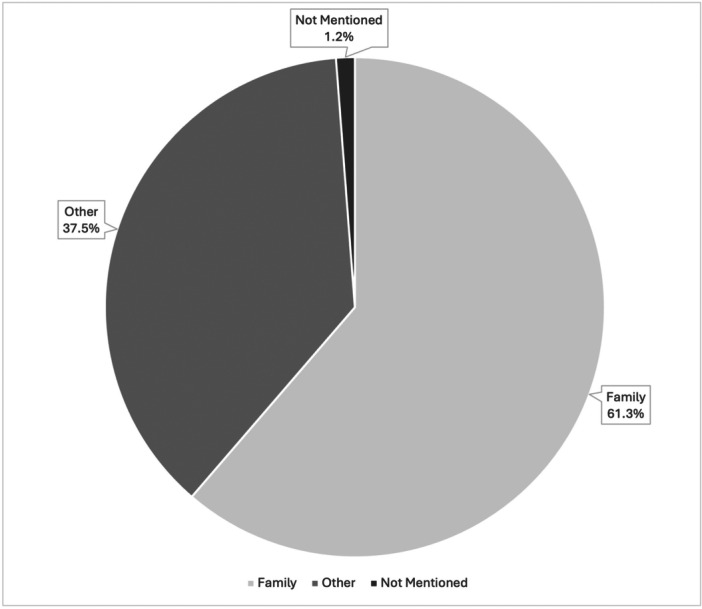
Patient relation to dog.

Patient relation to the dog was also of interest. Dogs were classified as family (lived in the same house as the patient), other (familiar dogs, strangers, etc.), or not mentioned (there was no mention of the relation in the report or patient chart). A total of 910 dogs were involved, as some patients were bitten by multiple dogs. The distribution of patient relation to dogs outlined in Figure 3 was: 61.3% family dogs (n = 558/910), 37.5% of other relation (n = 341/910), and 1.2% (n = 11/910) had no mention of patient relation. The fate of the dog following the bite was reported in 1 instance (n = 1/906, 0.1%).

A total of 1535 anatomical locations were injured during a dog bite. The 3 most injured locations were the cheeks (25.9%, n = 398/1535), lips (20.8%, n = 320/1535), and nose (9.0%, n = 138/1535). The number of injuries per anatomical location was organized by year in Table 4.

**Table 4:** Number of Injuries to Anatomical Location

Location	2012	2013	2014	2015	2016	2017	2018	2019	2020	2021	2022	2023	Total (%)
Cheek	10	29	24	31	20	29	42	51	38	35	45	44	398 (25.9%)
Lip	14	23	18	13	10	16	33	38	28	38	47	42	320 (20.8%)
Nose	4	10	9	1	16	3	11	18	15	13	20	18	138 (9.0%)
Additional injuries to other sites	22	4	6	12	4	11	11	15	14	15	19	20	133 (8.7%)
Eyelid	2	4	6	3	8	5	5	16	4	15	8	17	93 (6.1%)
Ear	4	3	7	10	8	7	9	5	11	5	7	9	85 (5.5%)
Forehead	2	4	6	6	5	3	4	9	12	7	7	12	77 (5.0%)
Chin	3	5	3	6	1	7	4	12	6	4	9	3	63 (4.1%)
Scalp	1	2	6	7	9	5	3	6	6	5	6	7	63 (4.1%)
Neck	2	3	2	1	5	5	4	11	7	6	6	4	56 (3.6%)
Eye	0	2	2	2	0	1	4	14	7	3	9	6	50 (3.3%)
Intraoral	1	3	2	1	2	0	0	1	3	2	4	2	21 (1.4%)
Eyebrow	0	0	0	2	0	2	2	0	1	4	1	3	15 (1.0%)
Temple	0	1	0	1	1	3	0	1	2	2	0	2	13 (0.8%)
Jaw	1	3	1	0	1	0	1	1	0	0	1	1	10 (0.7%)
Total	46	96	92	96	90	97	133	198	154	154	189	190	1535 (100%)

Of the 906 patients, 17.0% (n = 154) required hospital admission and 83.0% (n = 752) did not. Wounds were managed operatively, at bedside, or conservatively. There was a total of 912 management plans because some patients received multiple types of management. Regarding management, 16.9% (n = 153/906) were managed operatively, 65.9% (n = 597/906) were managed at bedside, and 17.9% (n = 162/906) were managed conservatively.

Following management of injuries, 96.9% (n = 878/906) of patients were prescribed antibiotics. A total of 1128 prescriptions were written in which amoxicillin‐clavulanate (n = 707/1128, 62.7%), ampicillin‐sulbactam (n = 197/1128, 17.5%), and clindamycin (n = 59/1,128, 5.2%) were the 3 most prescribed. All prescribed antibiotics were listed in Table 5.

**Table 5:** Antibiotics Prescribed to Patients With Dog Bites

Antibiotic	Number of prescriptions written (n) (%)
Amoxicillin‐Clavulanate	707 (62.7%)
Ampicillin‐Sulbactam	197 (17.5%)
Clindamycin	59 (5.2%)
Bacitracin	36 (3.2%)
Trimethoprim‐Sulfamethoxazole	21 (1.9%)
Cefazolin	15 (1.3%)
Ciprofloxacin	14 (1.2%)
Doxycycline	13 (1.2%)
Metronidazole	11 (1.0%)
Cefalexin	7 (0.6%)
Vancomycin	7 (0.6%)
Erythromycin	6 (0.5%)
Levofloxacin	6 (0.5%)
Ceftriaxone	6 (0.5%)
Moxifloxacin	5 (0.4%)
Cefuroxime	3 (0.3%)
Piperacillin‐Tazobactam	3 (0.3%)
Gentamicin	3 (0.3%)
Meropenem	2 (0.2%)
Amoxicillin	2 (0.2%)
Ceftazidime	2 (0.2%)
Azithromycin	1 (0.1%)
Linezolid	1 (0.1%)
Cefepime	1 (0.1%)

Vaccination status of the dog and patient were investigated. A total of 138 patients (15.2%) were bitten by a dog with unknown or outdated rabies vaccination status. Of these patients, only 35.5% (n = 49) received a rabies vaccination and 34.8% (n = 48) received rabies immunoglobulins. 254 patients (28.0%) were not current with their tetanus vaccination. However, only 78.7% (n = 200) received a booster.

## Discussion

Investigating dog bite injuries to the face, head, and neck is important since dog bites remain a public health concern.^5^, ^6^ This analysis of dog bite injuries over 11 years yielded pertinent information for clinicians of all disciplines. Our results showed most patients were under age 9. Most injuries involved a family dog and occurred while playing. The cheeks, lips, and nose were the most likely location for a bite to the face.

Dog bite injuries inflict physical and emotional damage. Patients with facial injuries have increased rates of anxiety, depression, and body image issues.^14^ A recent systematic review notes posttraumatic stress disorder and other psychological sequela being frequently reported in children who suffered dog bite injuries.^15^ Coupling this with public health concerns bite frequency, it is important to educate patients and families on preventing or recovering from dog bites to the face.

Results of our study show a gradual increase in facial dog bite injuries until 2020, when there was a noticeable decline in injuries and patients. The gradual increase aligns with data from the Center for Disease Control and Prevention (CDC). Per the CDC, fatal injuries from being bitten or struck by dogs doubled between 2018 and 2021 with further increase in the total number of bites.^16^ Similarly to our results, 1 study reports a decrease in incidence and severity of dog bites during 2020, associated with the lockdown portion of the pandemic.^17^ This is further supported by findings from another study that showed 79% of patients avoided seeking necessary medical care in 2020 due to fears over contracting the coronavirus.^18^

Our results found no major difference in time of year when dog bite injuries occurred. This differs from pre‐existing literature showing increased frequency of dog bites in the summer months.^19^, ^20^ Results of one study describe the greatest risk of dog bites within the summer months, though their findings also support an increased risk in fall and spring compared to winter. Additionally, this study reports higher frequency of dog bites on weekends compared to weekdays.^20^

Our cohort showed that young pediatric patients were most likely to be bit, with 58.3% being under age 9. These findings support previously published studies that prove increased incidence of head, neck, and face dog bite injuries in young children compared to adolescents, teenagers, and adults.^7^, ^8^, ^9^, ^10^, ^11^, ^12^, ^21^, ^22^, ^23^, ^24^, ^25^ One study specifically notes that periorbital dog bite injuries and combinations of periorbital, nose, lips, and/or cheeks are more common in children than adults.^24^ A systematic review of over 86,000 patients demonstrates that children under 9 years of age experience the greatest burden of injuries with children under age 6 years at greatest risk for serious injuries of the head, neck, and face.^26^ This higher propensity for craniomaxillofacial, head, and neck dog bites in children compared to adults can be attributed to the higher cranial mass to body ratio, especially in younger children.^13^, ^27^

Though there were 68 unique dog breeds represented in this study, the most common being Pitbulls, Labrador Retrievers, and German Shepherds. According to a 2023 article from the American Kennel Club (AKC), the Labrador Retriever and German Shepherd are the second and fourth most common dogs in the United States, respectively.^28^ Therefore, it is intuitive that the most common breeds are most commonly involved in dog bites. Interestingly, the AKC does not recognize the Pitbull as a breed because the club founders did not want fighting dogs to be associated with prized hunting dogs.^29^ The most common breed involved in biting humans differs between studies, but the Jack Russell Terrier, Pitbull, German Shepherd, Rottweiler, Doberman, and Husky have all been cited.^22^, ^30^, ^31^ One single‐site study notes that Pitbulls inflicted more complex wounds in their patient sample.^30^ Given there is no consensus in what breeds bite most often, it is important to educate patients on how to engage with dogs.

Our results show that patients were more likely to be bitten by a dog that lived within their home. This supports results from another study stating that children were more likely to be bitten by a familiar dog than an unknown dog.^31^ In fact, the reported incidence of being bitten by a familiar dog ranges anywhere from 25% to 83%.^22^, ^32^, ^33^ This increased incidence may be the result of patient interactions with the dog; more interactions with a dog means increased chances for a bite to occur. In addition, patients, specifically children, are more likely to interact with familiar dogs than strange ones. Depending on the interactions between the patient and the dog, there may be provocation for the dog bite.

Playing with the dog was the most reported provocation for an injury. Humans are imperfect at distinguishing between play and non‐play behaviors in dogs.^34^ Therefore, humans may not recognize the dog's intent, leading to annoyance in the dog and therefore a bite. Additionally, if the behavior of the dog changes during play, and humans are inaccurate at deciphering the intentions of the dog, the human could keep playing when the dog changes its mind, which could lead to a bite. One study shows that adults had difficulty reading signs of dog fear and anxiety in videos of dogs playing with children.^35^ Therefore, targeted education on dog behaviors is necessary to prevent dog bite injuries in patients of all ages.

Our cohort of patients had the highest number of injuries to the cheeks, lips, and nose. These findings are consistent with prior studies, and this is particularly relevant in pediatric patient populations.^7^, ^8^, ^9^, ^10^, ^11^, ^12^, ^13^, ^21^, ^22^, ^23^, ^24^, ^25^, ^26^, ^36^ Across all dog bite injuries, the face is the third most commonly bitten, with the upper and lower limbs being the most common.^13^ Given the shorter stature of younger children, their faces are closer to the mouth of a dog, which may explain the higher propensity for these injuries occurring in children versus adults. In addition, the cheeks being the most bitten site on the face could be due to the larger surface area compared to other facial structures.

Operative repair occurred in 16.8% of patients, bedside management was performed in 65.5% of patients, and 17.8% of patients were managed conservatively. Rates of operative repair in existing literature ranged from 7.4% to 16.9%.^25^, ^36^, ^37^ Previously established risk factors for operative repair include longer laceration length, involvement of eyelids, involvement of multiple facial regions, and severe injuries.^38^ Previously documented outcomes have no significant differences between patients receiving bedside versus operative repair.^38^, ^39^ However, 1 study cites higher infection rates in lacerations repaired by emergency room personnel.^39^ There is little data regarding efficacy of conservative management of dog bite wounds, but 1 case series reports 6 of 14 patients required minor scar revisions following conservative approaches.^40^

One study found immediate repair of dog bite injuries yielded better cosmetic results compared to delayed closure.^41^ However, a previous systematic review and meta‐analysis discovered no high‐certainty evidence supporting either primary or delayed closure over the opposing method.^42^ In the current study, cosmetic outcomes were not collected given that many patients were seen acutely in the emergency department without scheduled follow‐up.

In our study, 17.0% of patients required hospital admission for dog bite injuries. Infected wounds, complicated injuries, immunocompromised patients, previous evaluations for the same injury, and injuries to the head, upper extremity, or multiple anatomic locations have all been cited as risk factors for hospitalization following a dog bite.^42^ Our rates of admission were higher than a prior studies in which hospital admission ranged from 9.7% to 13.2%.^36^, ^37^

Since wound infections are the greatest risk factor for hospitalization, it is paramount to consider antibiotics in patients with dog bite injuries.^42^, ^43^ Dog saliva can expose patients to pathogens such as *Pasteurella multocida*, *Staphylococcus aureus*, *Viridans streptococci*, *Capnocytophaga canimorsus*, and oral anaerobes.^44^ Extended‐spectrum penicillins with beta‐lactamase inhibitors, such as amoxicillin‐clavulanate, offer the greatest coverage for these pathogens.^44^ In fact, a 3‐to‐5‐day course of amoxicillin‐clavulanate is considered first‐line antibiotic prophylaxis for dog bites.^43^ Clindamycin is a suitable alternative for patients with penicillin allergies.^43^ In our study, 96.9% of patients were prescribed antibiotics with a majority being prescribed amoxicillin‐clavulanate or its intravenous equivalent, ampicillin‐sulbactam.

Rabies vaccination and immunoglobulins are not indicated for every patient bitten by a dog. According to the CDC, patients should seek consultation of a healthcare provider following a bite, but those bitten by a dog with outdated or unknown rabies vaccination status of should start postexposure prophylaxis (PEP).^45^ Dogs can be monitored for 10 days prior to starting PEP as dogs have a shorter incubation time for the disease than humans.^41^ PEP varies based off prior exposure/rabies vaccination, but it can include up to 5 doses of vaccination and 1 dose of immunoglobulins.^41^ Based on our results, only 35.5% of patients received vaccination and 34.8% received immunoglobulins when indicated. However, many of these patients opted to observe the dog prior to starting PEP.

Tetanus vaccinations or boosters are essential following any traumatic injury. Though *Clostridium tetani* is not normal dog saliva flora, any puncture wound can introduce the bacterium into the body.^43^, ^46^ Current guidelines recommend updating tetanus vaccination or providing immunoglobulins after a puncture injury if the most recent vaccination was over 5 years prior, the patient is pregnant, or the patient has received less than 3 doses in their lifetime.^46^ According to our data, only 78.7% of patients received a booster when indicated. Interestingly, there are 5 cases reported in the literature of patients developing tetanus following a dog bite and proper tetanus PEP.^47^ Nonetheless, it is paramount to update tetanus vaccinations and educate patients on the importance of vaccination following puncture injuries.

Based on the results of our study and the current literature, there is room for improving dog bite prevention, particularly in the pediatric patient population. The AVMA has available materials on preventing dog bites, many of which center around recognizing patterns and changes in dog behavior.^48^, ^49^, ^50^ Actions such as dropping their tail, pulling back their ears, or stiffening their body can indicate worrisome feelings, though this varies between dogs.^49^ Collaboration between veterinarians and physicians to educate patients, parents, and dog owners on body language and behaviors of each dog is essential to prevent further increases in dog bite rates.

There are limitations to note. This study is a retrospective chart review performed at a single institution, limited by documentation in the electronic medical record. Therefore, data might not be generalizable to other populations. Additionally, results of this study relied on accurate reporting on the Pennsylvania Dog Bite Form. We found forms omitting key information including relation to dog, dog breed, and fate of the dog. These omissions create a source of bias since data was not consistently provided and may not paint a complete picture of dog bite demographics. Another major limitation is injury acuity. Many patients were seen for one encounter without follow‐up. Therefore, outcomes data regarding infection rates, rabies rates, or tetanus rates is incomplete. Regarding outcomes of repairs, most sutures used were absorbable given that most patients are seen acutely in the emergency department without follow‐up.

Future studies should analyze outcomes from surgically repaired injuries and associated sequela, specifically timing of repair, infection rates, scar formation, and need for additional treatments. Ideally, studies would include prospectively obtained, standardized measures to further quantify injuries. Our results also highlight an opportunity for quality improvement in how, when, and what data must be collected upon evaluation for dog bite injuries. Analyzing the impact of the COVID‐19 pandemic on dog bite injuries given the transient changes noted in our cohort is another potential direction for forthcoming studies.

## Conclusion

Dog bites to the head, neck, and face are common injuries, especially in pediatric populations. The most injured locations are the cheek, lip, and nose, with many patients requiring repair and antibiotics. Our institution had higher admission rates following dog bites compared to previous studies. Understanding demographics of these injuries is important to identify physical and socioeconomic burdens of disease and areas of targeted education/preventative interventions. Given that most patients were of pediatric populations and were bitten while playing with a dog, results of our study show opportunities for multidisciplinary patient education regarding safely interacting with dogs and recognizing warning signs of a bite. Veterinarians can educate pet owners on identifying aggressive behaviors/body language in their pets, whereas physicians can educate patients on interacting with dogs. Additional public health efforts can provide families with education about safe interactions with dogs to minimize bite injuries.

## Author Contributions

**Andrew J. Rothka**: conceptualization, investigation, writing—original draft, writing—review and editing; **Marly Aziz**: conceptualization, investigation, writing—original draft; **Katelyn P. K. Nguyen**: conceptualization, investigation, writing—original draft; **F. Jeffrey Lorenz**: conceptualization, investigation, writing—original draft, writing—review and editing; **Heather K. Schopper**: conceptualization, investigation, writing—original draft, writing—review and editing; **Jessyka G. Lighthall**: conceptualization, investigation, writing—original draft, writing—review and editing.

## Disclosures

### Competing interests

None.

### Funding source

None.
